# Prevalence of and Factors Associated With Incidental Chronic Kidney Disease in Patients Newly Diagnosed With Type 2 Diabetes Mellitus

**DOI:** 10.7759/cureus.79235

**Published:** 2025-02-18

**Authors:** Duha A Alidrisi, Haider A Alidrisi, Khulood A Reman, Ali M Hadi

**Affiliations:** 1 Clinical Pharmacy, College of Pharmacy, University of Basrah, Basrah, IRQ; 2 Diabetes and Endocrinology, Faiha Specialized Diabetes, Endocrine, and Metabolism Center, Basrah, IRQ; 3 Diabetes and Endocrinology, College of Medicine, University of Basrah, Basrah, IRQ

**Keywords:** chronic kidney disease (ckd), estimated glomerular filtrate rate, glycated hemoglobin, glycosylated haemoglobin (hba1c), newly diagnosed diabetes mellitus, urine albumin globulin ratio (uacr)

## Abstract

Objective: The objective of this study was to detect the prevalence of incidental chronic kidney disease (CKD) in patients newly diagnosed with type 2 diabetes (T2D).

Method: This was a cross-sectional study conducted from July 2023 to November 2024, at Faiha Specialized Diabetes, Endocrine, and Metabolism Center and Al-Rafidain Specialized Center in Basrah, southern Iraq. A total of 202 newly diagnosed drug-naïve T2D patients were included. The baseline clinical and biochemical characteristics for the patients at inclusion. CKD was diagnosed by measuring the estimated glomerular filtration rate (eGFR) and urine albumin creatinine ratio (UACR).

Results: The mean age of patients included in the study was 49.1±12 years. CKD was diagnosed in 68 (33.7%) patients based on GFR <60 mL/minute/1.73 m^2^ and/or UACR ≥ 30 mg/g. The CKD categories G1, 2, G3a, and 3b were prevalent in 71.3%, 24.2%, 3.0%, and 1.5%, respectively. For albuminuria, 31.2% had UACR 10-30 mg/g, 22.8% had UACR 30-300 mg/g, and 7.9% had UACR higher than 300 mg/g. A stepwise binary regression analysis showed that higher patients’ age and HbA1c levels were the factors that were significantly associated with CKD.

Conclusion: incidental CKD is prevalent in one-third of the newly diagnosed T2D. Early screening for CKD is highly recommended as it will affect overall management.

## Introduction

In Iraq, type 2 diabetes mellitus (T2DM) represents a significant burden, with the reported age-adjusted prevalence of T2DM ranging between 8.5% and 13.9% [1]. The insidious nature of T2DM often leads to mild, unnoticed symptoms that can persist for years. Consequently, many individuals are diagnosed only after complications have developed. Chronic complications of T2DM include cardiovascular diseases, diabetic neuropathy, diabetic retinopathy, diabetic nephropathy, infections, and severe peripheral arterial disease [2].

Diabetic nephropathy is a complex condition characterized by various pathophysiological mechanisms. The primary drivers include chronic hyperglycemia that causes structural and functional kidney changes leading to chronic kidney disease (CKD) [3]. The initiated metabolic, hemodynamic, inflammatory, oxidative stress, protein glycosylation, and fibrosis all contribute to renal injury [4]. Patients with T2DM experience glomerular hyperfiltration initially, followed by glomerulosclerosis due to sustained pressure changes and the activation of the renin-angiotensin-aldosterone system (RAAS) further promotes kidney damage through increased vascular resistance and fibrosis [3,5].

CKD is defined by either kidney damage or decreased kidney function for at least three months, characterized by albuminuria and reduced glomerular filtration rate [6]. Over 850 million individuals worldwide are affected by CKD, which translates to 10-15% of the global population. In the Western world, the main risk factor for CKD development is diabetes, which is present in 30-50% of CKD patients [7].

Low estimated glomerular filtration rate (eGFR) and high albuminuria are identified as risk factors for all-cause and cardiovascular mortality, independent of traditional cardiovascular risk factors such as hypertension and dyslipidemia [8]. In the late CKD stages, the progression of cardiovascular disease is accelerated in these patients leading to left ventricular hypertrophy; these patients have the highest mortality risk from cardiovascular complications [9].

Early detection of CKD is important for timely intervention and prevention of disease progression to end-stage renal disease (ESKD) or cardiovascular complications. Thus, screening for CKD in patients with T2DM is recommended using the urine albumin-to-creatinine ratio (UACR) and eGFR at the time of T2DM diagnosis and at least annually [10].

CKD remains significantly underdiagnosed and undertreated, despite advancements in clinical guidelines and treatment options, as there is a general lack of awareness about it among the public, healthcare professionals, and health authorities [11]. In a retrospective single-center study in Southern Iraq in 2009, proteinuria was found in only 6.6% of patients with T2DM [12]. In another study from northern Iraq, diabetic nephropathy was prevalent in about 14% [13]. In a cohort study, only 23% of CKD patients were correctly diagnosed by their physicians, while a significant number were diagnosed with other conditions like cancer (29%) and hypertension (82%) [14]. The asymptomatic presentation of early CKD leads to underdiagnosis, as most individuals remain unaware of their condition. Additionally, the association of CKD with multiple long-term conditions complicates recognition and timely intervention by healthcare practitioners [15]. Despite the understanding of CKD diagnosis and its complications in T2DM, less than 50% of patients receive the recommended care for CKD and it is predicted to be the fifth leading cause of death globally by 2040 [10].

In this study, we aimed to detect the prevalence of CKD in newly diagnosed patients with drug-naïve T2DM by active screening.

## Materials and methods

This was a cross-sectional study conducted from July 2023 to November 2024 at Faiha Specialized Diabetes, Endocrine, and Metabolism Center and Al-Rafidain Specialized Center in Basrah, southern Iraq. The study included treatment-naïve adult patients (18 years and above) newly diagnosed with T2DM. The patients were diagnosed as having T2DM if they met the American Diabetes Association (ADA) diagnostic criteria [16]. We excluded patients with pregnancy, febrile illness, urinary tract infection, liver disease like cirrhosis, and recent hospitalization.

Sample size

Based on the population proportion of T2DM, the sample size for this study was 196 patients. This was calculated by Cochran’s formula with a confidence interval (CI) of 95% (z-score 1.96) and an alpha value of 0.06. A total of 202 patients with newly diagnosed T2DM were included in the study with an age range of 23-85 years.

Clinical data

These data include age, gender, and history of hypertension. For each patient, we measured the office blood pressure using an automatic blood pressure monitor. Patients were considered to have hypertension if they were known to have hypertension and were on anti-hypertensive medication or had blood pressure equal to or higher than 140/90 mmHg on two occasions [17]. We considered a systolic blood pressure (SBP) of equal to or higher than 140 mmHg as uncontrolled and a diastolic blood pressure (DBP) of equal or higher than 90 mmHg as uncontrolled. For every patient, the body weight was measured in kilograms (kg) and height in meters (m) with bare feet and light clothes. Body mass index (BMI) was calculated with the formula: $\frac{Weight\ (in\ kg)}{Height^{2}\ (in\ meter)}$. The participants were considered obese if they had a BMI of 30 (kg/m^2^) based on the World Health Organization criteria of obesity classification.

 Laboratory data

We took 10 mm of blood from each patient after eight hours of fasting at presentation and divided it into two tubes (ethylene diamine tetra-acetic Acid (EDTA) and clot activator tube). The samples from the EDTA tubes were used for glycosylated hemoglobin (HbA1c) analysis by the ion exchanges high-performance liquid chromatography using the D-10 Hemoglobin Testing System (Bio-Rad Laboratories, Inc., Hercules, California, United States). The serum was separated from the clot activator tubes and analyzed with the COBAS INTEGRA® 400 PLUS system (Labservis LTD, Baku, Azerbaijan) for the measurement of serum glucose (mg/dl), creatinine (mg/dl), total cholesterol (TC), triglyceride (TG), high-density lipoprotein (HDL-C), and low-density lipoprotein (LDL-C). The eGFR was estimated by the equation of the Chronic Kidney Disease Epidemiology Collaboration (CKD-EPI) and reported as a mL/minute/1.73 m^2^ [18]. The eGFR values were further categorized as G1 (eGFR >90 mL/minute/1.73 m^2^), G2 (eGFR 60-89 mL/minute/1.73 m^2^), G3a (eGFR 45-59 mL/minute/1.73 m^2^), G3b (eGFR 30-44 mL/minute/1.73 m^2^), G4 (eGFR 15-29 mL/minute/1.73 m^2^), G5 (eGFR <15 mL/minute/1.73 m^2^) [19].

From all the included patients, freshly voided urine samples were collected in clean containers for microscopic and biochemical analysis. Urine samples that showed RBC >2/high-power field (HPF), WBC >2/HPF, and urine samples that were positive for nitrites were discarded. Urine albumin (mg) and creatinine (g) were analyzed by COBAS INTEGRA 400 PLUS. The UACR was calculated by dividing albumin concentration in milligrams (mg) by creatinine concentration in grams (g). Two UACR readings were done three months apart to confirm albuminuria. A UACR of less than 10 mg/g was considered normal. Abnormal UACR was categorized as A1 (<30 mg/g), A2 (30-300 mg/g), and A3 (>300 mg/g) [19]. CKD was diagnosed when the eGFR was <60 mL/minute/1.73 m^2^ and/or UACR was ≥30 mg/g.

Statistical analysis

Data were analyzed using IBM SPSS Statistics for Windows, Version 26.0 (2019; IBM Corp., Armonk, New York, United States). We summarized qualitative data as frequencies and percentages, and quantitative data as mean ± standard deviation (SD). A stepwise binary regression analysis was used for adjustment of the factors associated with the development of CKD in the study patients.

## Results

Table 1 summarizes the general characteristics of the participants. The mean age was 49.1 ± 12 years, with 68 (33.7%) aged 55 years and older, and 44.6% of the patients were male. The mean HbA1c at inclusion was 8.5 ± 2.1% and only 28.7% had an HbA1c less than 7%. Hypertension was presented in 60.9% of the patients. The mean GFR and UACR were 96.4 ± 19.2 mL/minute/1.73 m^2^ and 144.5 ± 748.1 mg/g, respectively.

**Table 1:** General characteristics of the study participants (N=202) HbA1c, glycosylated hemoglobin; FBG, fasting blood glucose; RBG, random blood glucose; HDL-C, high-density lipoprotein; LDL-C, low-density lipoprotein; SBP, systolic blood pressure; DBP, diastolic blood pressure; GFR, glomerular filtration rate; UACR, urine albumin-creatinine ratio

Variables	Values
Age (years), mean ± SD	49.1 ± 12.0
Age ≥ 55 years, n (%)	68 (33.7)
Sex (male), n (%)	90 (44.6)
BMI (kg/m^2^), mean ± SD	31.1 ± 4.4
BMI ≥ 30 kg/m^2^, n (%)	135 (66.8)
HbA1c (%), mean ± SD	8.5 ± 2.1
HbA1c <7%, n (%)	58 (28.7)
FBG (mg/dl), mean ± SD	185.5 ± 107.0
RBG (mg/dl), mean ± SD	232.1 ± 113.7
Total cholesterol (mg/dl), mean ± SD	192.4 ± 60.8
Triglyceride (mg/dl), mean ± SD	237.9 ± 300.7
HDL-C (mg/dl), mean ± SD	41.8 ± 12.6
LDL-C (mg/dl), mean ± SD	118.1 ± 42.5
SBP (mmHg), mean ± SD	140.1 ± 20.3
DBP (mmHg), mean ± SD	84.9 ± 13.2
Hypertension, n (%)	122 (60.9)
Uncontrolled SBP, n (%)	89 (44.1)
Uncontrolled DBP, n (%)	62 (30.7)
Uncontrolled BP, n (%)	102 (50.5)
Creatinine (mg/dl), mean ± SD	0.81 ± 0.2
GFR (mL/minute/1.73 m^2^), mean ± SD	96.4 ± 19.2
UACR (mg/g), mean ± SD	144.5 ± 748.1

**Figure 1:**
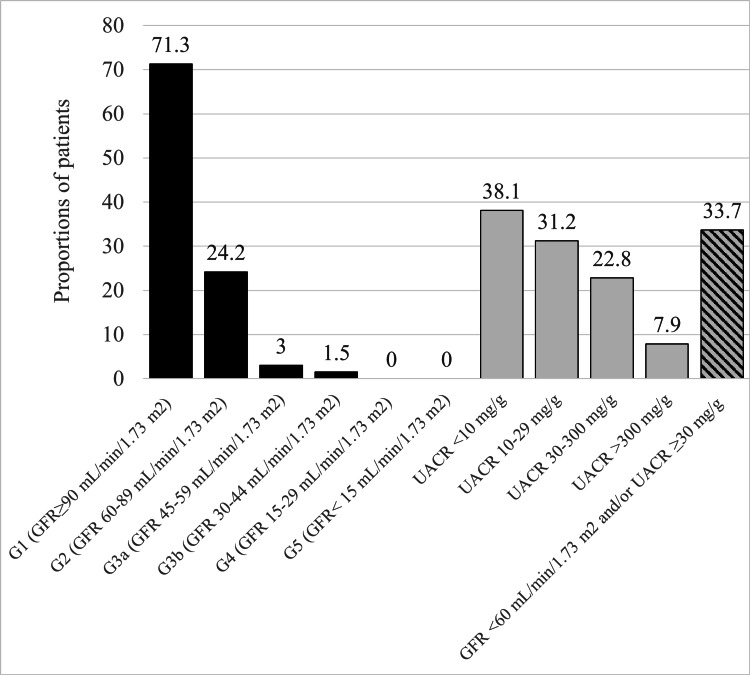
Prevalence of various categories of chronic kidney disease and albuminuria in the study participants (N=202) T2DM, type 2 diabetes mellitus; GFR, glomerular filtration rate; UACR, urine albumin-creatinine ratio

Sixty-eight (33.7%) participants had CKD based on GFR <60 mL/minute/1.73 m^2^ and/or UACR ≥ 30 mg/g. the CKD categories G1, 2, G3a, and 3b were prevalent in 144 (71.3%), 49 (24.2%), 6 (3.0%), and 3 (1.5%), respectively. None of the patients had CKD G4 or G5. For albuminuria, 77 (38.1%) had UACR <10 mg/g, 63 (31.2%) had UACR 10-30 mg/g, 16 (22.8%) had UACR 30-300 mg/g, and 16 (7.9%) had UACR >300 mg/g (Figure 1).

**Figure 2:**
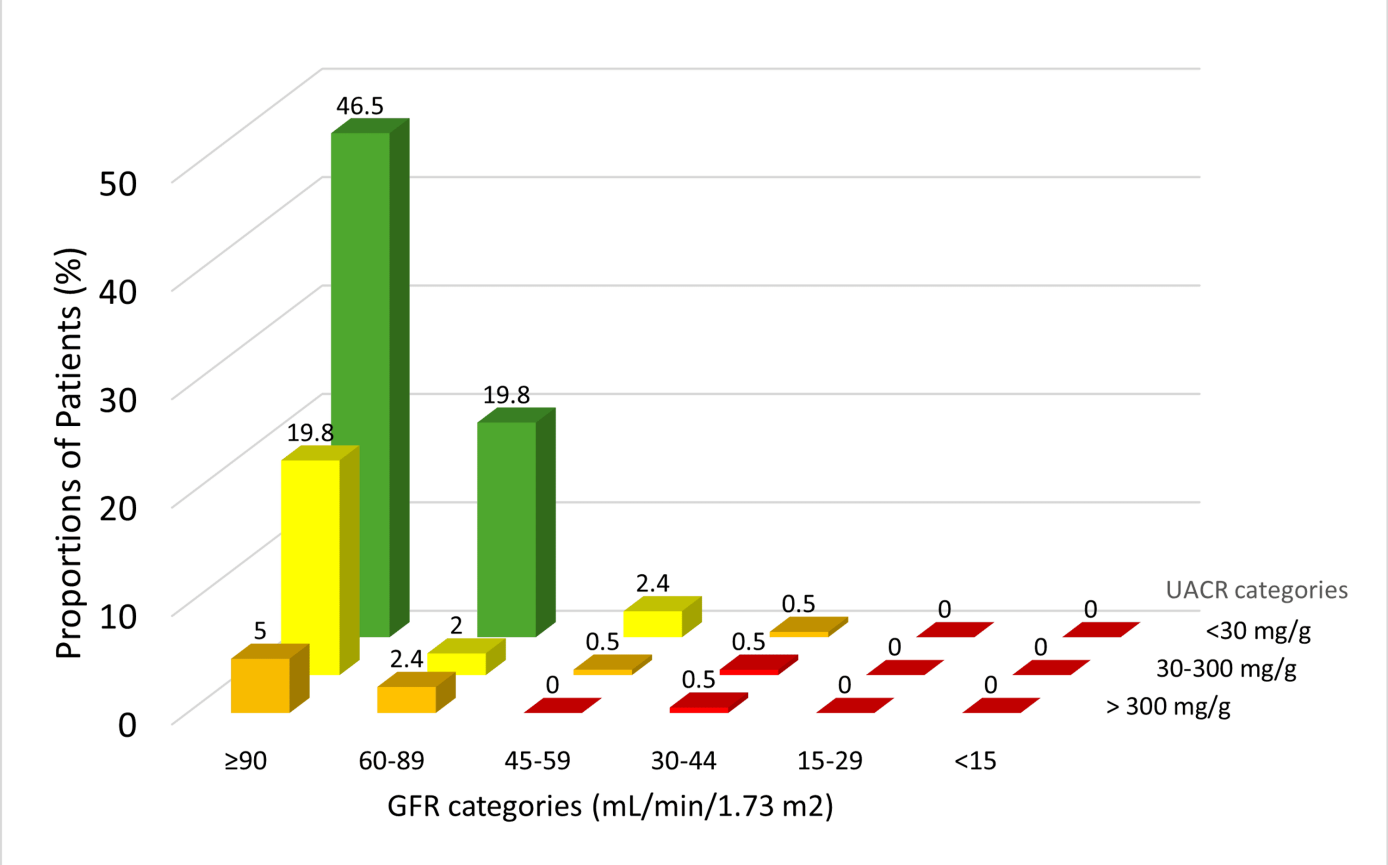
Heat map for future risk of cardiovascular and kidney complications in study particpants (N=202) Yellow: low risk; orange: moderate risk; red: high risk GFR, glomerular filtration rate; UACR, urine albumin creatinine ratio

Figure 2 shows the cross-tabulation of participants based on the GFR and UACR categories in the form of a “heat map” for future renal and cardiovascular complications. A total of 49 (24.2%) patients had CKD with low risk for future complications, 17 (8.4%) had moderate risk, and two (1.0%) had high risk.

A stepwise binary regression analysis was performed for adjustment of factors associated with CKD as shown in Table 2. The patients’ age and HbA1c levels were significantly associated with CKD. Increasing age was associated with an increased risk of having GFR <60 mL/minute/1.73 m^2^. Increasing HbA1c level was associated with an increased risk of having UACR of ≥30 mg/g and any CKD (GFR< 60 mL/minute/1.73 m^2^ and/or UACR ≥30 mg/g).

**Table 2:** Adjusted predictors for chronic kidney disease in study participants (N=202) S.E., standard error of the coefficient; HbA1c, glycosylated hemoglobin; UACR, urine albumin-creatinine ratio; BP, blood pressure; TC, total cholesterol; TG, triglyceride; HDL-C, high-density lipoprotein; LDL-C, low-density lipoprotein; GFR, glomerular filtration rate

Outcome	Variable	S.E.	P value	Odds ratio	95% CI
UACR ≥ 30 mg/g	Sex (male)	0.3	0.1	0.5	0.2 - 1.1
	Age	0.01	0.8	0.9	0.9 - 1.02
	BMI	0.04	0.06	0.9	0.8 - 1.006
	HbA1c	0.07	0.02	1.2	1.03 - 1.4
	Hypertension	0.4	0.9	1.003	0.4 - 2.3
	Uncontrolled BP	0.5	0.6	0.7	0.2 - 2.3
	TC	0.007	.9	1.001	0.9 - 1.01
	TG	0.001	0.6	1.001	0.9 - 1.003
	HDL-C	0.01	0.3	0.9	0.9 - 1.01
	LDL-C	0.008	0.9	1.0	0.9 - 1.01
GFR < 60 mL/minute/1.73 m^2^	Sex (male)	0.8	0.7	1.3	0.2 - 7.6
	Age	0.03	0.02	1.08	1.01 - 1.1
	BMI	0.09	0.1	0.8	0.7 - 1.05
	HbA1c	0.2	0.6	1.0	0.7 - 1.5
	Hypertension	1.1	0.07	7.5	0.8 - 67.3
	Uncontrolled BP	1.1	0.05	0.1	0.01 - 1.02
	TC	0.03	0.5	0.9	0.9 - 1.04
	TG	0.005	0.7	1.002	0.9 - 1.01
	HDL-C	0.05	0.8	1.009	0.9 - 1.1
	LDL-C	0.03	0.3	1.03	0.9 - 1.1
GFR < 60 mL/minute/1.73 m^2^ and/or UACR ≥ 30 mg/g	Sex (male)	0.3	0.4	0.7	0.3 - 1.5
	Age	0.01	0.4	1.01	0.9 - 1.03
	BMI	0.03	0.1	0.9	0.8 - 1.01
	HbA1c	0.07	0.01	1.2	1.03 - 1.3
	Hypertension	0.5	0.2	1.9	0.6 - 5.7
	Uncontrolled BP	0.5	0.6	0.7	0.2 - 2.2
	TC	0.007	0.9	1.000	0.9 - 1.01
	TG	0.001	0.5	1.001	0.9 - 1.003
	HDL-C	0.01	0.6	0.9	0.9 - 1.02
	LDL-C	0.008	0.8	1.0	0.9 - 1.0

## Discussion

In this study, one-third of the newly diagnosed T2DM patients had CKD, 4.5% had eGFR <60 mL/minute/1.73 m² and about 30% had UACR ≥30 mg/g. This implies that kidney damage can occur in the early periods of T2DM diagnosis. Furthermore, the possibility of remaining with undiagnosed T2DM for an extended period and the presence of other comorbidities like hypertension and dyslipidemia.

Many studies have evaluated the prevalence of CKD in early T2DM with varying designs. In a retrospective study in southern Iraq, the prevalence of CKD was only 6.6% based on proteinuria with two-fifths within the first five years of T2DM diagnosis [12]. This is a bit lower than the result of the current study. The importance of active screening for CKD as recommended and the underdiagnosis of CKD in our locality is shown in the current study. In a cross-sectional study in Saudi Arabia, the prevalence of CKD was about 45% in patients with a recent diagnosis of T2DM within six months [20]. In a large cohort study that included more than 36,000 patients with T2DM in the United States, the prevalence of CKD was 31.6% at T2DM diagnosis, and half of them had CKD stages G3-G5 [21]. In a retrospective study from Singapore including newly diagnosed T2DM patients, 7.9% had CKD at their first screening [22]. By the two-year follow-up, one-third developed CKD; albuminuria was responsible for the vast majority of CKD diagnoses (85% of total CKD). In a study from the Taiwan Diabetes Registry for determining the risk factors for early CKD stages in recently diagnosed T2DM within one year, 27.8% of participants were determined to have early CKD stages (G1-G3a), and again albuminuria was the major factor for the CKD diagnosis [23]. In the same study, patients were also categorized based on Kidney Disease: Improving Global Outcomes (KDIGO) for the risk of progression of kidney disease and cardiovascular complications, and 21.4%, 6.3, and 2.4% fell within the low, moderate, and high risk. These results are comparable to our results in which we found that about one out of each 10 newly diagnosed T2DM patients had moderate to high KDIGO risk. Results from the study by Chao et al. of patients with incident diabetes from the Longitudinal Cohort of Diabetes Patients (LCDP) cohort indicated that patients who later developed CKD had a significantly higher cardiovascular risk and remained so through the following years compared to those without CKD [24]. This finding explores the importance of active screening for early categorization to start the recommended management strategy.

In the current study, high HbA1c at baseline independently predicted incident CKD at T2DM diagnosis. Several clinical studies have highlighted the association between elevated HbA1c at the time of T2DM diagnosis and the risk of developing CKD [20,21,23,25,26]. A cohort study of adults with diabetes from 1988 to 2014 in the United States demonstrated that individuals with newly diagnosed T2DM and HbA1c >8.0% had a significantly higher rate of eGFR decline compared to those with HbA1c <7.0% [27]. In another population-based study from China, it was found that higher HbA1c was linked to albuminuria independently to hypertension and BMI [28]. The higher HbA1c at T2DM diagnosis factor may indicate undiagnosed and untreated hyperglycemia for years, and so longer duration of exposure to the pathogenic processes for the development of CKD.

In the current study, we found that the higher the age at T2DM diagnosis the higher the risk for CKD diagnosis. Some other studies have found a similar link between the age at T2DM diagnosis and incident CKD [21,23]. Individuals with early onset T2DM have higher lifetime CKD risk due to prolonged exposure to hyperglycemia which increases kidney damage over time [29]. Conversely, older newly diagnosed T2DM patients are also at increased CKD risk of age-related nephron loss rather than hyperglycemia alone. Additionally, older patients are more likely to experience other competing risks before kidney disease progression [30].

Some studies detected the association of other factors with incidental CKD in the form of abnormal lipid profile, hypertension, uncontrolled blood pressure, and BMI. In the current study, we did not find a significant association between these factors and CKD. Other studies found that abnormal lipid profiles predict incidental CKD [20,23,26]. On the other hand, hypertension and increasing BMI have been found to be independent predictors of incidental CKD [21,23,26] and increased CKD risk [21,26], respectively. These differences may be explained by the variability in the studies’ designs and the characteristics of the participants included in these studies.

Limitations

The study's cross-sectional design may not fully capture the effects of factors associated with incidental CKD. Diagnosing CKD using eGFR and UACR might underestimate its prevalence, especially in patients with higher eGFR (>60) and early kidney structural changes. Additionally, the inclusion of patients based on self-reported diagnosis of new T2DM rather than periodic screening raises concerns about undiagnosed T2DM.

## Conclusions

Incidental CKD was prevalent in 33.7% of newly diagnosed T2D patients based on eGFR and UACR. UACR was the major contributor to CKD diagnosis. Higher HbA1c and patients’ age could independently predict CKD diagnosis. Early detection of CKD using both eGFR and UACR is crucial for timely intervention to prevent disease progression and associated cardiovascular complications.
